# Associations Between Fathers’ Masculinity Orientation and Anticipated Reaction Toward Their Child’s Coming Out

**DOI:** 10.3389/fpsyg.2021.711988

**Published:** 2021-12-03

**Authors:** Dirk Kranz

**Affiliations:** Department of Psychology, University of Trier, Trier, Germany

**Keywords:** fathers, homosexuality, LG children, coming out (or disclosure), distress, rejection, acceptance

## Abstract

The present study examined associations between fathers’ masculinity orientation and their anticipated reaction toward their child’s coming out as lesbian or gay (LG). Participants were 134 German fathers (28 to 60years) of a minor child. They were asked how they would personally react if, one day, their child disclosed their LG identity to them. As hypothesized, fathers with a stronger masculinity orientation (i.e., adherence to traditional male gender norms, such as independence, assertiveness, and physical strength) reported that they would be more likely to reject their LG child. This association was serially mediated by two factors: fathers’ general anti-LG attitudes (i.e., level of homophobia) and their emotional distress due to their child’s coming out (e.g., feelings of anger, shame, or sadness). The result pattern was independent of the child’s gender or age. The discussion centers on the problematic role of traditional masculinity when it comes to fathers’ acceptance of their non-heterosexual child.

## Introduction

How would fathers react if their child came out as a lesbian or gay (LG) person? Given that less than 5% of the overall population feels sexually attracted to persons of the same sex (Bailey et al., 2016; Haversath et al., 2017), this scenario is not likely to happen but within the realm of possibility. We suggest that fathers with a traditional masculinity orientation tend to reject rather than accept an LG child. Such personality–behavior association should be mediated by two factors: anti-LG attitudes, as consequence of masculinity orientation, and the affective reaction toward the child’s coming out, as antecedence of the behavioral reaction. Before specifying our hypotheses, we briefly review the concepts and findings that are central to our study.

### Coming Out as an LG Child

For LG adolescents, disclosing their sexual orientation is a big biographical step (Savin-Williams and Cohen, 2015; Alonzo and Buttitta, 2019). They must decide on whether and when to come out, and, most precariously, with whom to share their LG identity. There is no comparable need for self-disclosure for heterosexual adolescents, as their majority sexual orientation conforms to the predominant heteronormative ideology. In general, self-disclosure is the “process by which individuals permit themselves to be known by others” (Taylor, 1979, p. 112). In this sense, “being known” is both a condition and consequence of the development of intimate relationships (Collins and Miller, 1994; Greene et al., 2006). Despite this, many LG people face, over and again, a *disclosure dilemma* (Griffith and Hebl, 2002). On the one hand, outness is conducive to social integration (Beals et al., 2009; Weisz et al., 2016); on the other, it bears the risk of discrimination (Legate et al., 2012; Riggle et al., 2017).

For most LG adolescents, coming out to their parents, their earliest and closest relationship, is a highly stressful experience. They fear their parents’ negative reactions, such as feelings of shame, guilt, sadness, disappointment, or anger, and, at the behavioral level, parents’ ignorance or denial of their child’s sexual orientation, or even parents’ rejection of their child as a person (LaSala, 2000; Heatherington and Lavner, 2008). How parents respond to their child’s sexual orientation is associated with a number of psychosocial outcomes. LG youth who report parental acceptance show higher self-esteem and well-being, whereas LG youth who report parental rejection show more internalizing and externalizing problems, including anxiety and depression, loneliness, substance abuse, sexual risk behavior, and suicidal thoughts and behavior (Rosario et al., 2009; Ryan et al., 2010; Puckett et al., 2015; Baiocco et al., 2016). Acceptance of their LG child is also positively associated with parents’ own psychosocial functioning (Connolly, 2005; Willoughby et al., 2010).

Against the backdrop of increasingly positive attitudes toward homosexuality in Western societies (Roberts, 2019; Takács and Szalma, 2020), an increasing number of LG adolescents come out at increasingly younger ages (Dunlap, 2016; Bishop et al., 2020). Overall, parents today show less rejection and more acceptance of their LG children than before (Beals and Peplau, 2006; Hank and Salzburger, 2015). That said, there is still considerable variability in parental reactions toward their child’s coming out (Rothman et al., 2012; Samarova et al., 2013; Martos et al., 2015; Rosati et al., 2020). Most research that examined influential factors related to parental reactions to their children’s coming out showed that the parent’s gender matters. Fathers are less likely to be told (Švab and Kuhar, 2014; Mitrani et al., 2017), less likely to be told first (Savin-Williams, 1998; Ryan et al., 2015), and more likely to react negatively than mothers (Boxer et al., 1991; D’Augelli and Hershberger, 1993; Ben-Ari, 1995; Maguen et al., 2002; Samarova et al., 2013; Charmaraman et al., 2021; but see also Hillier, 2002; D’Augelli et al., 2005). These gender differences are consistent with a body of research showing that men reject LG people more than women (Herek, 2002; Van den Akker et al., 2013).

### Masculinity-Homophobia Link

There is broad consensus among scholars that gender differences in homophobia (i.e., anti-LG attitudes and behaviors) are based on socially constructed and culturally mediated gender roles (Parrott et al., 2002; Goodman and Moradi, 2008; Herek and McLemore, 2013; Plummer, 2014). Gender roles are widely shared beliefs about attributes and behaviors of women and men that are normative for each sex (Eagly, 1987). The traditional male gender role is characterized by attributes like independence, competence, assertiveness, social dominance, risk proneness, and physical strength (Bem, 1974; Spence and Helmreich, 1979; Smiler, 2004). Men with such masculinity orientation tend to adhere to the traditional patriarchal family model (Bulanda, 2004; Petts et al., 2018). Accordingly, men should marry women and give their (then) wives children; fathers and mothers are expected to divide their family tasks as breadwinners and caregivers; and fathers should be the ultimate authorities in their families, as men should be in society in general (McAdams et al., 2008; Banchefsky and Park, 2016).

LG people are difficult to accept from the standpoint of traditional masculinity, as they transgress gender norms through their same-sex attraction alone (McCreary, 1994; Lehavot and Lambert, 2007). Many heterosexuals, especially men, perceive LG people as gender inverted: lesbians are thought to be more similar to heterosexual men than to heterosexual women, and gay men are thought to be more similar to heterosexual women than to heterosexual men (Kite and Deaux, 1987; Blashill and Powlishta, 2009). Such stereotypical thinking has a kernel of truth, as LG people indeed show less gender norm conformity (or, more gender norm flexibility) than heterosexuals (Lippa, 2005; Greaves et al., 2017). Therefore, the term *gender shift* describes LG reality more adequately than *gender inversion* (Lippa, 2008). That said, heterosexuals’ assumption of gender inversion all too often indicates anti-LG prejudice and disrespect, as common homophobic swear words reflect (e.g., *dyke* or *faggot*; Peel, 2005; Hegarty, 2006).

Given the pervasiveness of the gender inversion stereotype, a traditionally masculine oriented father whose child comes out as LG might easily conclude he has failed to raise his child to be a “real woman” or a “real man.” He might feel ashamed for his, from his point of view, gender nonconforming child. The coming out of a gay son might be especially challenging for a traditional father, as he might blame himself for not being an adequate role model in his son’s masculinity development (Levant, 2011; Horn and Wong, 2014). Correspondingly, some studies found that fathers react more negatively toward their son’s compared to their daughter’s coming out (D’Augelli, 2006; Baiocco et al., 2015), while other studies did not find this pattern (Savin-Williams and Ream, 2003; Pistella et al., 2020). Nevertheless, such gender differences would be consistent with the higher degree of homophobia that heterosexual men show toward gay men compared to lesbians (Steffens and Wagner, 2004; Morrison and Morrison, 2011).

Importantly, masculinity, as we use the term throughout this article, refers to *traditional* masculinity. Related, not identical, concepts are, from a critical sociological perspective, *hegemonic masculinity* (Connell, 1995), and *toxic masculinity*, with the latter concept emphasizing harmful anti-social (e.g., misogynous or homophobic) effects of traditional masculinity (Harrington, 2020). Traditional masculinity has consistently been shown to be closely related to homophobia, at both the attitudinal level (Parrott et al., 2002; Keiller, 2010; Harbaugh and Lindsey, 2015) and the behavioral level (Patel et al., 1995; Franklin, 2000; Birkett and Espelage, 2015). Although we recognize the recent discussion about other forms of *masculinity*, such as gay-friendly *inclusive* masculinity (Anderson and McCormack, 2018), these forms are not within the scope of our empirical work, but will be addressed in the Discussion section.

### Affect–Behavior Link

In general, emotions are feelings about something or someone of significance to us (our goals, values, desires, needs, etc.; Ellsworth and Scherer, 2003; Nussbaum, 2003). We are, for example, sad about something valuable we have lost or proud of our achievements or someone’s achievements who is close to us. Emotions include specific appraisals, such as judgments of valence, responsibility, and mutability (Weiner, 1985). Based on our phylogenetic and ontogenetic background, our personality and attitudes, emotions occur spontaneously and unintentionally, which has to be distinguished from secondary, deliberate attempts of emotion regulation (i.e., how we deal with our emotions; De Sousa, 1987).

Consider the father whose child discloses to him that they are LG. This father might response with sadness when fearing that his child could be bullied by their peers (Willoughby et al., 2006; Horn and Wong, 2014) or when anticipating that his child would not make him a grandfather, at least, not in a traditional setting (Baiocco et al., 2015; Jadwin-Cakmak et al., 2015). In increasingly LG-friendly societies, the father’s reaction could also be positive, especially, if he is an open-minded person and has a strong and warm connection to his child. He might, for example, be proud of his coming-out child when appreciating their striving for autonomy and authenticity; his pride might also refer to the high degree of closeness and trustfulness that characterizes the father-child relationship (Mena and Vaccaro, 2013; Perrin-Wallqvist and Lindblom, 2015).

Whether a father responds with sadness or pride, or any other negative or positive emotion, toward his child’s coming out, might primarily depend on his overall attitude toward homosexuality (Cramer and Roach, 1988; Holtzen and Agresti, 1990; Ghosh, 2020). An LG hostile father might respond with negative affect (i.e., the amalgam of negatively valenced emotions; Russell, 2003), an LG friendly father, however, with positive affect. Importantly, negative and positive affect are not mutually exclusive. That is, the father’s affective response can be emotionally ambivalent (Cohler, 2004; Van Bergen et al., 2020). He might feel, for example, at the same time sadness about the discrimination his child could face as a member of a sexual minority and pride about his child’s self-confidence to come out with their sexual identity. Generally speaking, mixed emotions convey ambivalent information about one’s current state of relationship with the (social) world (Larsen et al., 2001; Russell, 2017).

Besides the informative function (Schwarz, 1990; Izard, 2011), many, but not all, emotions have a motivational function (Frijda, 2004; Zeelenberg et al., 2008). That is, emotions not only indicate the significance of personal goals (values, desires, needs, etc.) in a given situation, they also prioritize and energize goal striving. This motivational function has been mainly discussed in terms of evolutionary efficiency. Accordingly, the duality of positive and negative affect (or, pleasure and pain) is fundamentally linked with approach and avoidance behavior, respectively (Higgins, 1997; Elliot, 2006). The enormous variety and flexibility of human emotion and action are thought to build upon this duality (Plutchik, 2001; Nesse and Ellsworth, 2009).

Regarding the father whose child discloses their LG sexual orientation to him, a positive affective response might be primarily associated with acceptance, whereas a negative affective response might be primarily associated with rejection of his child. Reflecting the approach–avoidance duality, acceptance and rejection have repeatedly been confirmed as the fundamental parental reaction patterns toward their child’s coming out at the behavioral level (D’Augelli et al., 1998; Rohner et al., 2005; Fuller, 2017). In cases of emotional ambivalence, acceptance and rejection motivations may well occur together and provoke approach-avoidance conflicts (Emmons and King, 1988; Corr and Krupić, 2017).

### Hypotheses

Based on the outlined masculinity-homophobia and affect-behavior links, we propose the following two hypotheses:

*Hypothesis 1:* The stronger fathers’ traditional masculinity orientation, the more negative their anticipated behavioral reaction toward their LG child’s coming out will be (i.e., more rejection and less acceptance of their child).

*Hypothesis 2:* This effect can be explained, firstly, by masculine fathers’ higher level of anti-LG attitudes, and, secondly, their more negative affective reaction toward their child’s coming out (i.e., more negative and less positive emotions).

**Figure 1:**
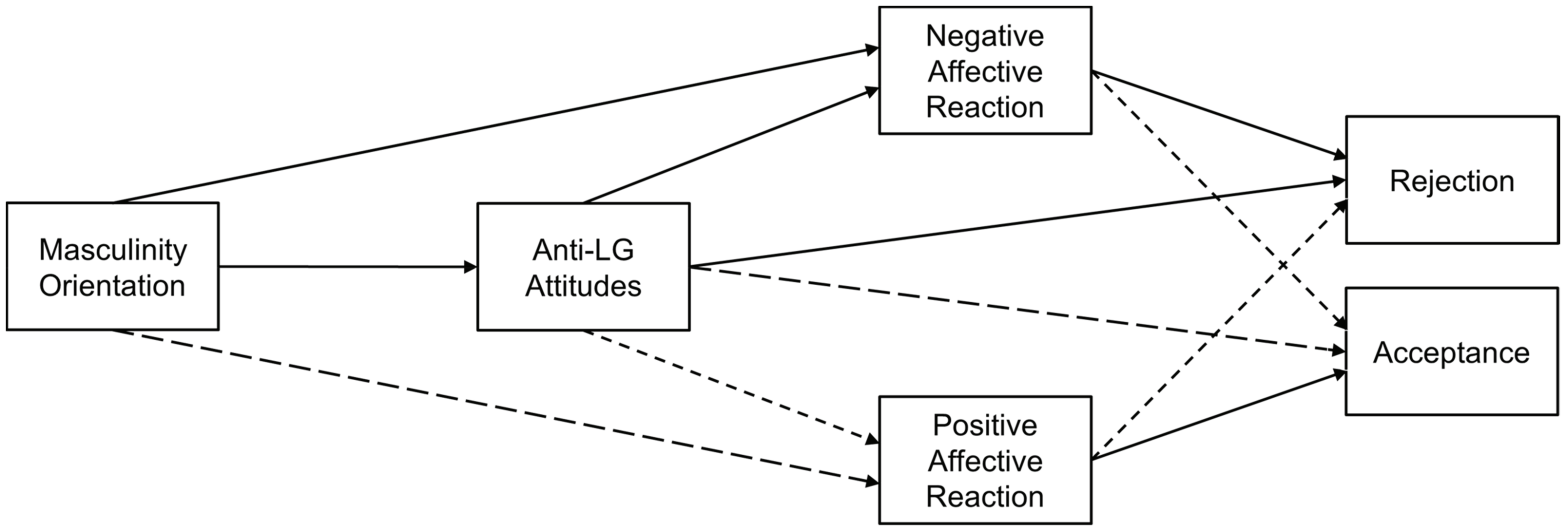
Conceptual mediation model for the association between masculinity orientation and anticipated reactions toward the child’s coming out. Dashed and solid paths indicate hypothesized negative and positive associations, respectively.

Methodologically speaking, Hypothesis 1 postulates a personality-behavior link between masculinity orientation and LG child rejection/acceptance, which Hypothesis 2 then breaks down into a combined serial and parallel mediation. Anti-LG attitudes are suggested as first-order mediator, negative and positive affective reaction as parallel second-order mediators. The conceptual model in Figure 1 summarizes our hypotheses. It implies negative and positive affect as well as rejection and acceptance as two-dimensional constructs, reflecting the possibility of affective and behavioral ambivalence, respectively.

## Materials and Methods

### Sample

Participants were *N*=134 German fathers with an age range of 28 to 60years (*M*=40.86, *SD*=6.60). Almost all fathers self-identified as heterosexual (*n*=132; *n*=2 bisexual fathers), were either married (*n*=116) or in a committed relationship (*n*=14; *n*=2 fathers in divorce), and employed (*n*=129; *n*=4 unemployed, *n*=1 student). Participants had one to four children (*M*=1.96, *SD*=0.83), including, at least, one minor child. Regarding the youngest child, about whose possible coming out the fathers were asked to reflect, the vast majority was in early and late childhood (i.e., prepubertal; <6years: *n*=97; 6–11years: *n*=22); only a minority were adolescents (12–18years: *n*=11; *n*=4 children without age information). The gender of the youngest child was relatively balanced (*n*=71 girls, *n*=59 boys, *n*=4 children without gender information).

### Procedure

The present study was conducted online. Fathers could participate if they had, at least, one minor child. A kindergarten and a primary school located in southwest Germany supported sample recruitment by providing children’s fathers with a study information letter including the online study link. In agreement with the Ethical Principles of the German Psychological Society (Deutsche Gesellschaft für Psychologie, 2016), this voluntary and anonymous study did not require the approval of an ethics committee. Participants were broadly informed about the research question before they consented to participate (“How do fathers think about developmental paths their children might take?”). Upon request, by sending a separate e-mail message (to preserve anonymity), participants were informed about the specific research purpose and results when the study had been completed.

### Measures

Participants’ main task was to “imagine that, one day, your youngest child will disclose to you that she is lesbian or he is gay. What do you think, how will you personally react to your child’s coming out?” Participants were purposely asked to focus on their youngest child. Imagining that one’s child could be LG should be easier when the child is still young and, probably, has not yet discovered or disclosed their sexual orientation. The affective and behavioral reaction items that participants completed upon this question were derived from the relevant literature cited above and recent reviews thereof (Chrisler, 2017; Fuller, 2017; Ghosh, 2020). Furthermore, participants were asked about their general masculinity orientation and attitudes toward homosexuality. Finally, they provided demographic information.

#### Affective Reaction

Fathers’ anticipated affective reaction toward their child’s coming out was measured with an adjective list, consisting of 10 negative and 6 positive items, to be rated on a 5-point scale (from *would not apply to me at all* to *would totally apply to me*). A principal axis analysis (oblimin rotation, eigenvalue>1) confirmed a two-factor solution, accounting for 63.5% of the variance. Due to poor factor loadings (main loadings<0.60 and/or cross-loadings>0.40; Comrey and Lee, 1992), we had to exclude one positive item (compassionate). Two other positive items (calm and serene) loaded inversely on the negative affect factor and, thus, were, after recoding, assigned to the negative affect scale, which then comprised 12 items (e.g., ashamed, guilty, angry, sad), while the positive affect scale comprised only 3 items (relieved, happy, and proud). Scale reliabilities were excellent and sufficient, respectively, Cronbach’s alphas=0.94 and 0.78.

#### Behavioral Reaction

Fathers’ anticipated behavioral reaction toward their child’s coming out was measured with 6 items, 3 negative items (rejection of the child, ignorance and denial of the child’s sexual orientation^1^) and 3 positive items (acceptance, care, and support of the child), to be rated on a 5-point scale (from *would not apply to me at all* to *would totally apply to me*). A principal axis analysis (oblimin rotation, eigenvalue>1) suggested a one-factor solution, explaining 49.4% of the total variance. Therefore, after recoding the negative items, all items were aggregated to one reliable rejection scale, Cronbach’s alpha=0.78. Because this measure was highly positively skewed (right-skewed distribution), we additionally applied a normalizing log10 transformation (Tabachnick and Fidell, 2007). Results of all analyses remained practically the same, irrespective of whether we used the transformed or untransformed measure. For ease of presentation and interpretation, the results we report below are based on the untransformed rejection measure.

#### Masculinity Orientation

We extended McCreary et al.’s (2005) Masculinity Scale to measure participants’ traditional masculinity orientation. Four of the originally five items combined two or, in one case, three statements (e.g., “Do you believe that taking risks that are sometimes dangerous is part of what it means to be a man and part of what distinguishes men from women?,” “As a man, how important is it for you to control your emotions and never to reveal sadness or vulnerability?”). To facilitate item processing, these items were split up into separate, short single-statement items, giving a 10-item masculinity measure. Each item was scored on a 5-point scale (from *not at all true* to *absolutely true* or, depending on the item wording, from *not at all important* to *absolutely important*). High scores indicate a more pronounced masculinity orientation. Scale reliability was sufficient, Cronbach’s alpha=0.74.

#### Anti-LG Attitudes

We measured participants’ anti-LG attitudes with Falomir-Pichastor and Mugny’s (2009) Attitudes Toward Homosexuality Scale (short form by Anderson et al., 2018). The 16 items reflect a variety of contemporary pro- and anti-LG attitudes (e.g., “I am in solidarity with LG people,” “I would be embarrassed if a gay person made sexual advances toward me”). We excluded one item (“It would not bother me at all if my child was LG”) to avoid semantic overlap and, thus, artificial associations with the measures related to the child’s coming out. Items were rated on a 7-point scale (from *totally disagree* to *totally agree*). High scores indicate a more negative attitude toward LG individuals (i.e., a higher degree of homophobia). Scale reliability was excellent, Cronbach’s alpha=0.94.

## Results

As a preliminary step, we inspected scale means and bivariate correlations, the latter with a main interest in associations between fathers’ traditional masculinity orientation, anti-LG attitudes, and anticipated reaction toward the child’s coming out. We then conducted the crucial mediation analysis to test whether the association between masculinity orientation and LG child rejection was mediated by anti-LG attitudes and the affective response toward the child’s coming out. Note that the statistical model we tested slightly differed from the conceptual model described in the Section “Introduction” (*cf.* Figures 1 vs. 2). Since we could not identify separate rejection and acceptance factors (the factor analysis rather suggested a one-factor solution), the unified rejection (vs. acceptance) scale served as the only outcome measure at the behavioral level.

**Figure 2:**
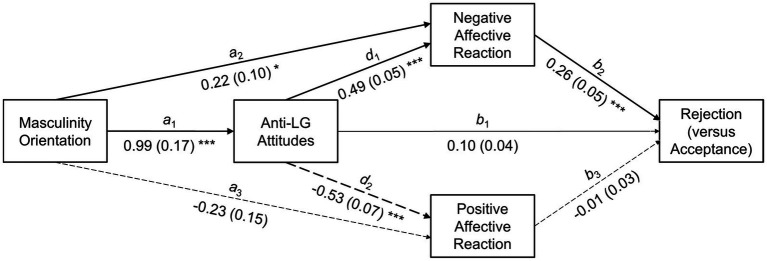
Statistical mediation model for the association between masculinity orientation and anticipated reactions toward the child’s coming out. Dashed and solid paths indicate statistically significant negative and positive associations, respectively. Thin paths indicate non-significant associations. Path coefficients are unstandardized regression weights (*B*s); their standard errors (*SE*s) are in parentheses. ^*^p<0.05, ^**^p<0.01, and ^***^p<0.001.

### Preliminary Analysis

Descriptives and correlations of the personality and reaction measures are shown in Table 1. The average masculinity and positive affective reaction scores were close to the middle of the scale range, whereas the average homophobia, negative affective reaction, and child rejection scores were (substantially) below the middle of the scale range.

**Table 1:** Bivariate correlations and scale characteristics.

Path	MASC	ALGATT	NEGAFF	POSAFF	REJECT
MASC	(0.74)	0.46^***^	0.46^***^	−0.38^***^	0.27^**^
ALGATT		(0.94)	0.76^***^	−0.63^***^	0.62^***^
NEGAFF			(0.94)	−0.57^***^	0.68^***^
POSAFF				(0.78)	−0.40^***^
REJECT					(0.78)
Possible range	1.00–5.00	1.00–7.00	1.00–5.00	1.00–5.00	1.00–5.00
Observed range	1.70–4.50	1.00–6.20	1.00–4.58	1.00–5.00	2.67–5.00
*M*	3.14	2.71	1.77	2.83	1.52
*SD*	0.54	1.17	0.85	1.08	0.42

*MASC, Masculinity orientation; ALGATT, anti-LG attitudes; NEGAFF/POSAFF, negative/positive affective reaction; REJECT, LG child rejection. Scale reliabilities (Cronbach α) are on diagonal in parentheses*.

^*^p<0.05,

** *p<0.01, and*

*** *p<0.001*.

As expected, participants’ masculinity orientation correlated positively with their anti-LG attitudes. Both variables were substantially related to negative and (with opposite sign) positive affective reaction toward the child’s coming out and, furthermore, to child rejection. Not surprisingly, negative and positive affective reaction were inversely interrelated and also showed substantial relationships with LG child rejection.

Except for a comparably weak but statistically significant relationship between participants’ age and masculinity orientation (*r*=−0.19, *p*=0.028), there were no significant correlations between demographics (fathers’ age and number of children, gender, and age of their youngest child) and personality variables (masculinity orientation and anti-LG attitudes) or reaction variables (affective reaction and child rejection). Therefore, we did not consider demographics as relevant covariates in the following mediation analysis.

### Mediation Analysis

Results of the mediation analysis are presented in Figure 2 (simple path coefficients) and Table 2 (coefficients for the mediated pathways). Paths were named after Baron and Kenny (1986): *a*, *b*, and *c* paths refer to predictor-mediator, mediator-outcome, and predictor-outcome associations, respectively, while *d* paths refer to inter-mediator associations. The mediation analysis involves testing a total effect (*c*, mediators excluded), a direct effect (*c*′, mediators included), and a total indirect effect (all indirect effects summed up). The total effect is the sum of the indirect effects (or, the total indirect effect) and the direct effect. Complete mediation occurs if the total effect can be completely explained by the mediators. Mediation can occur without a significant total effect, when parallel mediators operate in opposite directions.

**Table 2:** Path coefficients for the mediation model.

Effect	Path	*B*	*SE*	*LLCI*	*ULCI*	*p*
Total	*c*	0.21	0.06	0.12	0.31	0.001
Direct	*c*'	−0.07	0.05	−0.15	0.01	0.173
Indirect (Total)		0.28	0.06	0.19	0.41	<0.001
(1) MASC → ALGATT → REJECT	*a*_1_ × *b*_1_	0.10	0.06	0.01	0.20	0.082
(2) MASC → NEGAFF → REJECT	*a*_2_ × *b*_2_	0.06	0.03	0.02	0.14	0.005
(3) MASC → POSAFF → REJECT	*a*_3_ × *b*_3_	−0.00	0.01	−0.02	0.01	0.338
(4) MASC → ALGATT → NEGAFF → REJECT	*a*_1_ × *d*_1_ × *b*_2_	0.13	0.05	0.07	0.24	<0.001
(5) MASC → ALGATT → POSAFF → REJECT	*a*_1_ × *d*_2_ × *b*_3_	−0.01	0.01	−0.03	0.01	0.481

*MASC, Masculinity orientation; ALGATT, anti-LG attitudes; NEGAFF/POSAFF, negative/positive affective reaction; REJECT, LG child rejection. Bs and SEs are unstandardized regression weights and their standard errors; LLCIs and ULCIs are the lower and upper limits of the 95% confidence intervals for the unstandardized regression weights, based on a bootstrapping procedure*.

Specifically, traditional masculinity orientation was expected to affect LG child rejection through five indirect pathways: (1) through anti-LG attitudes alone (*a*_1_×*b*_1_), (2) through the negative affective reaction alone (*a*_2_×*b*_2_), (3) through the positive affective reaction alone (*a*_3_×*b*_3_), (4) through anti-LG attitudes and negative affective reaction sequentially (*a*_1_×*d*_1_×*b*_2_), and (5) through anti-LG attitudes and positive affective reaction sequentially (*a*_1_×*d*_2_×*b*_3_). Indirect effects (1) to (3) reflect simple mediation, while indirect effects (4) and (5) reflect serial mediation. Considered together, indirect effects (2) and (3) as well as indirect effects (4) and (5) each reflect—simple and serial, respectively—parallel mediation.

Combined serial and parallel mediation was tested with a structural equation framework, based entirely on observed variables (Hayes et al., 2017). A nonparametric bootstrapping procedure was applied (Preacher and Hayes, 2008). It involved computing unstandardized indirect effects for each of the 5,000 bias-corrected bootstrapped samples and calculating the 95% confidence interval (CI) values. Before conducting the mediation analysis, we checked for multicollinearity among the predictor and mediator variables. All tolerance values were above 0.36, exceeding the cut-off point of 0.10 (Cohen et al., 2003), which suggested that multicollinearity was not an issue here.

Reflecting the bivariate correlation, there was a total positive effect *c* of traditional masculinity orientation on LG child rejection. The direct effect *c*′, however, was no longer significant when anti-LG attitudes and the affective reaction toward the child’s coming out were added to the model, indicating mediation. A closer look at the specific indirect effects showed that the negative affective reaction was the crucial mediator. It fully mediated the association between masculinity orientation and child rejection as both a simple mediator (*a*_2_×*b*_2_) and a serial mediator (*via* anti-LG attitudes; *a*_1_×*d*_1_×*b*_2_). The positive affective reaction, however, did not link the association between masculinity orientation and child rejection, neither as a simple nor a serial mediator. Furthermore, masculinity-driven anti-LG attitudes did not serve as a first-order mediator; the inclusion of negative affect as second-order mediator was necessary to explain the masculinity-rejection association.

Nevertheless, anti-LG attitudes linked, in the sense of simple mediation, the associations between traditional masculinity orientation and both negative and positive affective reaction toward the child’s coming out (*a*_1_×*d*_1_ and *a*_1_×*d*_2_). That is, anti-LG attitudes established the positive association between masculinity orientation and the negative affective reaction (*B*=0.50, *SE*=0.09, 95% CI [0.35, 0.65], *p*=0.001) as well as the negative association between masculinity orientation and the positive affective reaction toward the child’s coming out (*B*=−0.52, *SE*=0.10, 95% CI [−0.71, −0.37], *p*=0.001).

The amounts of variance explained by the combined serial and parallel mediation model were as follows: 20.8% for anti-LG attitudes, 58.8% for negative affective reaction, 40.4% for positive affective reaction, and, most importantly, 49.7% for rejection. That is, half of the variance in fathers’ rejection of their LG child could be explained by the variables considered in the model. All amounts of variance explained were statistically significant at *p*<0.005.

To examine the directionality of indirect effects, we also tested a reverse model with the negative and positive affective reaction to the child’s coming out as parallel first-order mediators and anti-LG attitudes as the second-order mediator. Except of the impact of masculinity orientation on LG child rejection through negative affect (a simple mediation effect already found in the original mediation model), none of the simple or serial mediation effects were significant, which additionally corroborated our mediation hypothesis.

## Discussion

Fatherhood is a long neglected field in family research, but there is growing awareness of the important role that fathers play in the development of their children (Lamb, 2010; Pleck, 2010). Our study examined associations between fathers’ traditional masculinity orientation (i.e., adherence to male gender norms such as independence, assertiveness, and physical strength) and their anticipated reaction toward their child’s self-disclosure as an LG person. The sample consisted almost exclusively of heterosexual fathers (two bisexual exceptions) of, at least, one minor, mostly prepubertal child. Hypothesis 1 stated that the more important it is to the father to be a traditionally masculine man, the more rejection and less acceptance of their LG child he should show. According to Hypothesis 2, this association should be mediated by two factors: the masculine father’s higher degree of anti-LG attitude (or, homophobia) and his more negative and/or less positive affective response toward their child’s coming out. The hypotheses were based on the theoretically grounded and empirically validated links between masculinity and homophobia as well as, more generally, between affect and behavior. They included the possibility of both affective and behavioral ambivalence.

The data confirmed negative and positive affective reaction toward the child’s coming out as separate factors. At the behavioral level, however, negative and positive reaction could not be empirically disentangled. Rejection and acceptance built one common factor. This pattern is not necessarily inconsistent with the existing literature. Compared to negative and positive affect (Watson et al., 1988; Carver, 2001), the two-factorial structure of rejection and acceptance seems less robust (Rohner and Cournoyer, 1994; Gomez and Rohner, 2011). Thus, we had to slightly change the mediation model by replacing the original two criteria of acceptance and rejection by the single criterion of rejection, with its opposite pole of acceptance on the same continuum.

As expected, the overall association between masculinity orientation and LG child rejection was significantly positive. Furthermore, fathers who were traditionally masculine oriented anticipated having more negative emotions (or, more emotional distress), if their child would come out as an LG person. Only one of the two pathways included in our model mediated this association. Specifically, masculinity was positively related to anti-LG attitudes, which were, as expected, inversely related to negative and positive affect upon the child’s coming out. Only negative affect, however, showed a significant (positive) relationship with rejection, while positive affect showed no analogous (negative) relationship. In other words, traditionally masculine oriented fathers were more homophobic and, therefore, reacted with more emotional distress, and, as a consequence, more rejection of their LG child.

Why did only negative affect, but not positive affect, mediate masculinity orientation, homophobia, and LG child rejection? Emotion researchers generally attribute more behavioral relevance to negative compared with positive emotions. This *negativity bias* (Baumeister et al., 2001; Rozin and Royzman, 2001) is mostly explained from an evolutionary perspective. Accordingly, negative emotions signal that something is wrong (in the worst case, life threatening); they mobilize mental and bodily resources that are necessary to manage (or survive) the situation (Nesse and Ellsworth, 2009). Positive emotions, however, are less activating; they signal safety and satisfaction (for their functional value, see Fredrickson, 2013). Referring to the negativity bias, it makes sense that fathers’ negative affect upon the child’s coming out is more closely related to rejection than positive affect and, thus, serves as a mediator of this relationship. Negative affect signals fathers with a traditional worldview that something is wrong with the LG child – a deviance that impedes child acceptance.

Although the main research interest of this study was on structure-oriented analysis, namely, the mediated association between masculinity orientation and LG child rejection, findings of level-oriented analysis are also instructive and important. Overall, fathers showed a moderate level of traditional masculinity orientation and a low level of homophobia. Both participants’ negative as well as positive affect upon their child’s coming out were somewhat restrained. Strikingly, fathers’ tendency to reject their LG child was extremely low. Taken together, these findings might reflect that Germany has become an LG friendly country over recent decades (Pew Research Center, 2013; European Commission, 2019). This trend should not be taken for granted, given the persecution of (primarily male) homosexuality during the Nazi regime, but also in the 1950s and 1960s (at least in West Germany; Moeller, 1994; Plant, 2011). Fathers’ overall tendency to accept rather than reject their LG child might also reflect general change in male parenting. Today, a generation of “new” fathers strives for the gender equalization of parenting norms and attempts to fulfill the originally maternal ideal of caregiving or involved parenting as well as the originally paternal task of breadwinning (Marsiglio and Roy, 2012; Dermott, 2014). This father generation strongly desires to build, maintain, and defend emotionally close relationships with their children, even when times are challenging.

Our study underlines the differentiation between level-oriented and structure-oriented analysis in the field of homophobia. Although fathers’ absolute scores of general homophobic attitudes and specific homophobic reactions toward their child’s coming out were low, homophobic processes instigated by traditional masculinity orientation were corroborated. In sum, findings are consistent with significant pro-LG change at the societal macro level, while, at the same time, they draw attention to ongoing patterns of homophobia at the micro level. Regarding prevention of and intervention in the family context, it is therefore important to identify societal groups in which traditional (or, even, toxic) masculinity and anti-LG prejudice are still tolerated or even reinforced. For example, LG children growing up in fundamentalist religious or chauvinist nationalist contexts, especially when mixed up with patriarchal ideology, are at a very high risk to face extreme forms of homophobia, including being abandoned by their families and becoming victims of hate crimes (Barnes and Meyer, 2012; Gibbs and Goldbach, 2015; Hirai et al., 2018; Adamczyk and Liao, 2019).

One important element of programs against paternal homophobia should be the introduction into masculinities in the plural (Connell, 1995). Masculinity does not necessarily have a homophobic face. Other LG-friendly forms combine, for example, physical strength with emotional sensitivity; they endorse intimate friendships among men and apply assertiveness to reject any form of discrimination against sexual minorities (Anderson, 2005). Another element of programs against paternal homophobia should be informed perspective taking (Todd and Galinsky, 2014): What if my own daughter or son were LG? *Reflecting* about this possibility *and knowing* that sexual orientation is no matter of parental (or any other social) influence or personal choice might make fathers more LG friendly. Gender differences in homophobia (for cross-cultural invariance, see Bettinsoli et al., 2020), including differences between mothers and fathers (for a review, see Heatherington and Lavner, 2008), suggest that men struggle more with such perspective taking than women. A third element should be the promotion of positive images of LG-friendly fathers. Recently, a well-known New Zealand center-right politician and long-serving MP, Nick Smith, apologized for having voted against same-sex marriage after his son came out as gay. “The error is all the more personal with my 20-year-old son being gay,” Smith said in his last speech in Parliament before retiring (Neilson, 2021).

### Limitations and Outlook

Our research is not without limitations. To the best of our knowledge, we investigated fathers’ anticipated response to their child’s coming out for the first time. In other words, this study was a pilot project, and as such its main limitation was sample size. It was at the lower end for mediation analysis (Fritz and MacKinnon, 2007; Schoemann et al., 2017). Therefore, future studies are needed to examine the stability of results. Some effort should be made to develop finer-grained measures of affective and behavioral parental reactions toward the child’s coming out (or, to adapt and extent existing measures, e.g., the Perceived Parental Reactions Scale by Willoughby et al., 2006, or the Parental Acceptance and Rejection of Sexual Orientation Scale by Kibrik et al., 2018, also based on the LG child perspective). These measures might also attempt to disentangle rejection (or, inversely, acceptance) of the child from rejection of the child’s sexual orientation. Regarding the last point, parental rejection of the child as a person might be associated with more detrimental consequences for the parent–child relationship as well as the child’s (and the parents’) well-being, compared to parental rejection of the child’s sexual orientation. Given the results of the present study, however, it is questionable whether the differentiation between the child as a person and their sexual orientation can be empirically supported. In our data set, parental ignorance and denial of the child’s sexual orientation loaded on the same factor as parental rejection of the child (and, with opposite signs, the child acceptance items). In other words, sexual orientation seems to be an essential and integral part of personality; parents who reject their LG child’s sexual orientation also disapprove of their child as a person.

Another limitation might be the hypothetical scenario used. We asked fathers about their affective and behavioral reactions if, one day, their child disclosed their LG identity to them. Affective and behavioral forecasting, however, are not free of bias (Warshaw and Davis, 1985; Wilson and Gilbert, 2005). To our knowledge, previous research on parental reactions toward their child’s coming out has been conducted entirely in retrospect; that is, parents or children were asked to remember their parents’ reactions (Fuller, 2017; Ghosh, 2020). Interestingly, similar biases occur when people predict and remember emotional situations (e.g., tendencies to overestimate or overreport, respectively, emotional intensities; Levine et al., 2018). Retrospective designs thus do not necessarily outperform prospective designs. That said, an optimum design would be prospective longitudinal. Correspondingly, a future study should follow a cohort of children and their fathers over a period of time (e.g., from late childhood to early adulthood), in order to determine, in the relevant subsample, how fathers really react toward their children’s coming out. At least, the baseline measurement should include measures of fathers’ masculinity orientation and anti-LG attitudes. Such research would allow for testing the (as we think and show with the present data, plausible) causal link between fathers’ masculinity orientation and their behavioral reaction to their child’s coming out, mediated by homophobia and affective reaction.

A third limitation concerns research focus. Future research might extend it in several ways. Participants were asked how they would react toward their youngest child’s coming out. Correspondingly, the target child was mostly younger than 6years. On the one hand, imagining that the child could be LG should be easier when they are still far away from starting their sexual life. On the other hand, children at such young age are completely dependent on and in very close relationships with their parents, which might provoke biased responses. Future studies might therefore direct attention to older children. They might additionally include mothers as participants and include measures of both masculine and feminine gender role orientation. By doing so, we could answer to what extent parents’ gender and/or gender role orientation predict LG child rejection (Conley, 2011). Future research might also address reactions toward the child’s coming out among other family members like siblings or grandparents, depending on their gender role orientation and attitudes toward homosexuality (Finkenauer et al., 2004). Finally, our focus was on LG coming out. Parents’ reactions toward their child’s coming out with other sexual orientations (e.g., bisexuality) and/or gender identities (e.g., transgender; Parent et al., 2013) certainly deserves attention in future studies.

A fourth limitation concerns research context. The present study was conducted in Germany and needs to be replicated in other countries. Public opinion on the acceptance of homosexuality remains sharply divided by country. People in Western countries show generally more acceptance of homosexuality than those in less developed and wealthy economies (Poushter and Kent, 2020). The association between traditional masculinity and homophobia, however, seems to be closer in Western countries than in other parts of the world (Bettinsoli et al., 2020). Societal changes also concern parenting. Given the increasing trend toward *new fatherhood* (Lamb, 2000), both participants’ ideal and reality of involved parenting might be considered in future research on parental reactions toward the child’s coming out.

## Conclusion

The present study investigated factors that influence how fathers think they would react if their child disclosed their LG identity to them. Factors impeding LG child acceptance were, in this order, traditional masculinity orientation, anti-LG attitudes, and negative affect (or, emotional distress) upon the child’s coming out. The parsimonious mediation model explained about half of the variance in LG child rejection. Although reflecting on having an LG child might be hypothetical, it is not without impact. Given that “what if” thoughts generally have a preparative function (Epstude et al., 2016), such reflections might predict fathers’ real reactions should the scenario come true. Moreover, and perhaps more importantly, if fathers have a child who is “still in the closet,” this child might be very sensitive to their father’s thinking and talking about sexual orientation issues. Referring to the Thomas theorem (situations defined as real are real in their consequences; Thomas and Thomas, 1928), LG children’s perception of their fathers’ attitudes about LG people and, in particular, about having an LG child, might have a tremendous impact on children’s relationship with and coming out to their fathers.

## Data Availability Statement

The statistical code and dataset, including all item wordings and response formats, are available at: https://dx.doi.org/10.23668/psycharchives.4779 and https://dx.doi.org/10.23668/psycharchives.4780, respectively.

## Ethics Statement

Ethical review and approval was not required for the study on human participants in accordance with the local legislation and institutional requirements. The participants provided their informed consent to participate in this study.

## Author Contributions

The author confirms being the sole contributor of this work and has approved it for publication.

## Funding

The publication was funded by the Open Access Fund of Trier University and the German Research Foundation (DFG) within the Open Access Publishing funding program.

## Conflict of Interest

The author declares that the research was conducted in the absence of any commercial or financial relationships that could be construed as a potential conflict of interest.

## Publisher’s Note

All claims expressed in this article are solely those of the authors and do not necessarily represent those of their affiliated organizations, or those of the publisher, the editors and the reviewers. Any product that may be evaluated in this article, or claim that may be made by its manufacturer, is not guaranteed or endorsed by the publisher.

## Footnotes

^1^A note on item wording: In German, one can reject (*ablehnen*) ones child, but ignoring (*ignorieren*) or denying (*verleugnen*) ones (minor) child as a person would reflect child neglect – which is a criminal category. Therefore, we worded the items assessing negative behavioral reaction as follows: “I would reject my child,” “I would ignore my child’s sexual orientation,” and “I would deny my child’s sexual orientation.” Compared to the German term *jemanden ablehnen*, the English term *to reject someone* has a wider meaning, including *disapproving of someone* (*jemanden zurückweisen*), but also more extreme forms of *breaking off* (*mit jemandem brechen*) and *abandoning someone* (*jemanden verstoÃƒÅ¸en*), aspects that are not covered by the German term *ablehnen*.
